# Academic Debate: Publications Which Promote Political Agendas Have no Place in Scientific and Medical Journals, and Academics Should Refrain from Publishing in Such Journals

**DOI:** 10.5041/RMMJ.10178

**Published:** 2015-01-29

**Authors:** Shimon Glick, A. Mark Clarfield, Rael D. Strous, Richard Horton

**Affiliations:** 1 Faculty of Health Sciences, Ben-Gurion University of the Negev, Beer-Sheva, Israel;; 2 Medical School for International Health, Ben-Gurion University of the Negev, Beer-Sheva, Israel;; 3 Beer Yaakov Mental Health Center, Beer Yaakov, Israel;; 4 Department of Psychiatry, Sackler Faculty of Medicine, Tel Aviv University, Tel Aviv, Israel; 5 Editor-in-Chief, The Lancet, London, UK

**Keywords:** Debate, medical journal, politics, publication ethics, scientific journal

## Abstract

This paper presents the full debate held on October 1, 2014, which focused on the following resolution: “Publications which promote political agendas have no place in scientific and medical journals, and academics should refrain from publishing in such journals.”

The debate moderator was Professor Shimon Glick. Taking the pro stance was Professor A. Mark Clarfield; the con stance was held by Professor Rael D. Strous. Following the first part of the debate, Dr Richard Horton, Editor-in-Chief of *The Lancet*, gave his thoughts on the topic. This was followed by the opportunity for rebuttal by Professors Clarfield and Strous. The debate was summarized and closed by Professor Glick.

This paper provides a slightly edited text of the debate, for ease of reading.

## DEBATE INTRODUCTION: SHIMON GLICK, MD

I would like to thank the organizers of this visit by Dr Richard Horton, editor of *The Lancet*, for inviting me to moderate the debate this morning on this important, relevant, and sensitive subject; thereby encouraging a spirited, respectful, and academic discussion of a difficult controversy that has arisen as a result of the recent letter published by *The Lancet*.

We will not follow the all too frequent Israeli custom in debates, which includes shouting, interruptions, and having both speakers talk simultaneously. Instead in deference to our guest we will try to follow a local modification of the rules governing the great traditions of the Oxford and Cambridge debates.

We have also selected two protagonists who are natives of the British Empire, one from South Africa and the other from Canada. I must emphasize clearly that the sides were assigned to the debaters at random, and the positions they will be espousing may not represent their actual opinions on the subject. Therefore, if you disagree with the position presented by a speaker, do not make your opposition personal; because it may well be that the speaker actually agrees with you fully.

Each speaker will be given 20 minutes for an initial statement and then 10 minutes for rebuttal. We will then permit audience participation by *short* questions addressed to the particular speaker in a respectful manner appropriate to an academic discussion. The rules will be enforced strictly. Unlike some debating societies, we will not have an official judging of the outcome of the debate, nor will an audience vote be held.

The resolution to be debated reads as follows:

Resolved that publications which promote political agendas have no place in scientific and medical journals and academics should refrain from publishing in such journals

The first speaker, supporting this resolution, will be Professor A. Mark Clarfield who heads the Medical School for International Health at Ben-Gurion University of the Negev.

His opponent will be Professor Rael Strous, Deputy Director of the Beer Yaakov Mental Health Center and Professor of Psychiatry of the Sackler Faculty of Medicine at Tel Aviv University.

## PRO STANCE: A. MARK CLARFIELD, MD, FRCPC

Good morning, *boker tov*, *saalam aleikum*.

I offer my most profound thanks to the Rambam Hospital, to the organizers of today’s debate, to Professor Shimon Glick for agreeing to take on the challenging task of moderating this event, and above to all to my esteemed opponent and colleague Professor Rael Strous, who has so graciously agreed to being beaten this morning.

Ladies and gentlemen, please allow me to repeat the resolution:

*A medical publication which promotes political agendas has no place in scientific and medical journalism, and academics should refrain from publishing in such journals*.

I have been asked to speak in favor of this resolution, and overall I will make two arguments:

1. First, indeed, a medical journal should stick *primarily* to medical issues as that is its expertise, that of its editors, that of its peer reviewers, and, above all, that of its readers.
2. Second, when a journal does deal with medical issues that *touch* on a political *aspect* (for example the relative size of a national health budget versus that of education), this publication must do so in a fair, disinterested, and unbiased manner, and must scrupulously avoid any hint of a conflict of interest. I will return to these terms: “fair,” “disinterested,” “unbiased,” “conflict of interest.”

A journal that breaks these rules (or is even *suspected* of doing so, and I will come back to this concept as well) will suffer a serious and well-deserved blow to its credibility. I need not remind us all that, in academia, intellectual and scientific integrity is really our only true currency.

I turn now to two related questions:

1. What is the difference between a medical journal and one which deals with politics?
  and
2. What is the harm in traversing this boundary?

To begin, I offer two examples of interesting clinical questions which I am confident will help clarify my point:

1. Should we screen for lung cancer in smokers?
  and
2. From my own field of geriatrics, are anti-dementia drugs (e.g. donepezil or rivastigmine—the latter having been discovered right here in Israel) indicated in the treatment of Alzheimer disease?

Let us now play a mind experiment and submit an article on either of these two subjects to say, *Foreign Affairs* or *International Political Science Review*. What would happen?

Professor Strous, I can promise you one thing. The editor would send these manuscripts back posthaste with a polite note suggesting that the authors must have erred; that they might well consider submitting to a *medical* journal.

And why would the editor take such a step? Clearly, because neither the journal’s editors nor its peer reviewers would have the expertise to judge the technical or scientific aspects of these submissions, nor would the journal’s readers be able to understand most of the arguments offered.

Alternatively, let us think of a purely *political* question which interests me deeply and is relevant to all of us in this room … Yes, I have it! The Two-State Solution as a way to end the Israel-Palestinian conflict. Clearly, any lasting way out from our 100-year-old conflict would be beneficial, especially to the health (in its broadest senses; see the World Health Organization (WHO) definition) of both peoples, not to mention to the economy, culture, environment, and a host of other benefits to our little contested patch of land here in the dusty Middle East.

Now back to my submission which I will entitle: “The Two-State Solution Is the Only Way to End the Israel-Palestinian Conflict: An Evidence-based Approach” (which as a personal aside I still believe in strongly as the best way forward). However, I would hardly think of sending such a submission to the *Journal of the American Medical Association* (*JAMA*), *The Lancet*, or even to the *Israel Medical Association Journal*. Appropriately, it would be rejected by the respective editors.

Another issue of great relevance to humanity, including health, is global warming. All of us (except of course for card-carrying members of the Flat Earth Society) will agree that this phenomenon is a true danger, not just on the medical side but to the very existence of our species. *What* could be more relevant to health in its widest sense?

On this issue let us indulge in another thought experiment: Let us conjure up an examination of the comparative costs to society of wind, solar power, hydro, coal, nuclear energy, and gas; and *voila*! we find that such a study has indeed just appeared. In fact, Charles Frank from the Brookings Institution recently published such a piece (as reported in *The Economist*). This paper found, counter-intuitively at least to my eyes, that wind and solar power are actually the most expensive forms of energy production—even when one takes the carbon footprint (or lack thereof) into the calculations. (By the way, he concluded that natural gas is the most cost-effective.)

To remind us all, we are talking about two issues:

First, a medical journal should stick to medical issues as that is its expertise, that of its editors, peer reviewers, and, above all, its readers.

And why shouldn’t Dr Frank submit this study to a medical journal? Is global warming not a health concern? The answer regarding submission would of course be “no,” since as physicians we do not have the *technical* expertise nor education to deal with this issue *professionally*. As educated citizens reading *The Economist*—yes—but as *experts* in our field, of course, no.

What about my second point, relating to the expectations and requirements of a medical journal when dealing with issues on the border between politics and medicine? Again, as pointed out earlier, when such issues are addressed:

A journal … must do so in a fair, disinterested, and unbiased manner, and must scrupulously avoid any hint of a conflict of interest.

To address this second condition, let us modify our thought experiment; for this case, Dr Frank’s findings now support coal mining as the cheapest form of energy production. But this pronouncement would and should make the critical reader think twice. What if it were later discovered that Dr Frank had been a long-standing advocate of the coal mining industry? What if the author had clearly hidden his connections or, perhaps better phrased, his “friendliness” to this industry?

Even worse, what if the journal editor had been aware of these conflicts but, because of his or her own personal opinions (perhaps he or she grew up in Wales or West Virginia or was now living in the state of Wyoming), that editor had forged ahead and arranged a fast-track publication? And what if he or she allowed the submission to be published without any counter-arguments as in a critical editorial or a “face-to-face” counterpoint? Of course, on being challenged, the editor might then offer a wishy-washy excuse that a few letters to the editor had been belatedly allowed, some con and, but of course to be fair, pro as well?

This would surely make one think, *n’est-ce pas*?

What if, under the guise of the topic’s relevance to “public health,” such a published paper ended up supporting coal as the cheapest and *healthiest* form of energy production? This would surely make one think and wonder about the editor’s intentions.

But let us now leave such a purely non-medical issue and move on to one in which not only do medicine and politics actually intersect but where medical expertise would indeed be relevant to the question at hand. To that end, I would like to introduce two Summary Statements.

### First Summary Statement: *Medical journals should stick primarily to their expertise and to the subjects their readers expect them to address*

With respect to a pertinent example let us examine the role of “Big Pharma” in modern medical care which of course is legitimate grist for a medical journal—at least for certain aspects of the question. This choice will also allow me to focus more on the second subject I have promised to address: the absolute need for fairness and the avoidance of potential conflicts of interest.

Another thought experiment: let us argue that, overall, Big Pharma does society more good than harm when it markets “me-too” drugs. Let us posit an American medical journal tackling this complex issue but one in which the editor consistently only publishes one side—that of Big Pharma. In addition, what if the authors of a major contribution to the field “forgot” to declare a relevant conflict of interest such as honoraria or speaker’s fees from the pharmaceutical industry?

Furthermore, what if it became clear that this Yankee editor were a card-carrying member of the Tea Party or even just an enthusiastic fellow traveler?

When all of this came out, Professor Strous, what would, or could, or should the editor of a medical journal do?

I contend, Sir, that there are only two options:

1. Since we all know that prevention is better than cure, a reputable medical journal with a diligent editor would and should have taken great care to avoid letting such a thing happen in the first place.
2. However, as Alexander Pope wrote in a famous poem in the seventeenth century, “To err is human, to forgive divine;” such slip-ups will inevitably occur. When they do, the scrupulous editor, being a well-educated gentleperson, will of course swiftly apologize. Not only will he or she say, “I am sorry, I erred,” but, even more importantly, the editor will make some serious amends.

However, if the editor refused to do so, one might well question either their good judgment, or wonder about their basic sense of fair play, or, even more seriously, be concerned about both.

### Second Summary Statement: *When medical journals do touch on the political aspects of medicine, of which there are many, they must tackle this with the utmost care and do so fairly and judiciously*

In this context I would like to return to a purely political issue and ask what the role of a medical journal might be in some issues I have picked at random from the news over the last few days:

1. The conflict between Russia and the Ukraine
2. China’s long-standing invasion and subsequent occupation of Tibet and cultural repression of its people
3. The UK has been in the news of late, especially with the recent referendum over Scottish Independence; what about the UK’s 400 year occupation of Catholic Northern Ireland (the natives call it Ulster) by Scottish Protestant settlers
4. The civil war raging in Syria just a few kilometers from Israel’s northern border, not very far from this morning’s debate

Of these four let us concentrate on the Ukrainian issue, as time does not allow me to go into these other tragic conflicts. What would a reputable medical journal have to say about this terrible conflict which has killed thousands to date and shows no real signs of abating?

We all have long known that war is not good for all living things, so simply repeating this mantra would not be particularly helpful. Again, this issue is not within the purview of a respectable medical journal. In fact, I believe we all might be just a tad surprised were we to see a “letter” condemning Ukrainians in general and Ukrainian doctors in particular by a group of Russian medical professionals masquerading as being concerned for the poor treatment of Russian speakers within the Ukraine.

Here again, with some justice we might wonder about the editor’s judgment or sense of intellectual fair play.

Of course this is a moot point; I don’t even know why I brought it up! A quick scan of a group of journals belonging to the International Committee of Medical Journal Editors (ICMJE) (e.g. *New England Journal of Medicine* (*NEJM*), *Canadian Medical Association Journal* (*CMAJ)*, *The Lancet*, etc.) revealed no such missive, nor is such a one likely to be published in the near future.

So far, I have clearly established that a respectable medical journal should *not* unilaterally promote a specific *political* agenda; this as opposed to dealing fairly—*fairly*, I repeat, Professor Strous—with the political aspects of relevant medical issues, to which it should indeed from time to time devote some of its scarce page resources.

However, when a medical journal is seen to clearly flout these rules, what should academics of good will do?

First, in deference to the good Irish colonel, I would *not* (I repeat, *not*) call for a formal *boycott* of such a publication. This would go against all of the rules and the *true* spirit of academic freedom which must always be avidly and courageously defended by all people of good will.

Distinguished members of the audience, and my feisty opponent Professor Straus, you might find yourselves surprised by my next statement, but I truly believe it: nor would I call for a *formal* campaign to oust the editor of a journal which flouted these rules of objectivity, fair play, proper declaration of conflicts of interest (of both authors and editors), and simply keeping to the journal’s medical mission.

Rather, this is clearly the publisher’s job; although he or she might be impressed by legitimate complaints from readers and customers or other commentators protesting a particular editor’s practices. As we all know, not dealing with episodes of editorial malpractice will only be detrimental to that journal’s academic reputation. In the end, this is its only true currency—since of course money, power, and prestige never enter the equation when we are talking about our own intellectual pursuits!

Interestingly, one need not reinvent the wheel as there are agreed-upon rules and guidelines clearly spelt out which might help a publisher in such a situation. These can be found in the statements of the above-mentioned ICMJE to which many top-notch journals belong (again: *NEJM*, *JAMA*, *CMAJ*, *The Lancet*, and, if I am not mistaken, about 12 others). For example, among several proclamations is their 2013 statement, entitled: “Recommendations for the Conduct, Reporting, Editing, and Publication of Scholarly Work in Medical Journals.”^1^

To wit:

Public trust in the scientific process and the credibility of published articles depend in part on how transparently conflicts of interest are handled during the planning, implementation, writing, peer review, *editing*, and publication of scientific work.^1^ [Emphasis added]

The statement continues, and I quote:

A conflict of interest exists when professional judgment concerning a primary interest … [such as] the validity of research may be influenced by a secondary interest …^1^

It is important to point out, and I quote again that:

Perceptions of conflict of interest [*perceptions!!*] are as important as actual conflicts of interest.^1^

These guidelines are meant for authors, peer reviewers, and editors. But specifically, with respect to the last-mentioned, the rules are crystal clear:

Editors who make final decisions about manuscripts should recuse themselves from editorial decisions if they have conflicts of interest or relationships that pose *potential* conflicts related to articles under consideration.^1^ [Emphasis added]

Now why, Professor Strous, *did* I take this little detour? I think the policies are clear enough and that a reputable publisher would of course do the right thing once it was pointed out in a calm, civil, and civilized manner that one of their editors (even a brilliant, talented, telegenic, and charismatic one) had transgressed. That being said—and I repeat for clarity—in academia there is no place for a *formal* boycott, even if we are dealing with what we perceive as a conscious or unconscious bias, or even (God forbid) a lack of intellectual good will.

So what can the lonely academic do when faced with a clear example of bias in academic publishing, a clear lack of fair play, and a sloped playing field?

I offer four alternatives:

1. As alluded to above, one would first try suasion. There is nothing like a civilized and open dialogue such we are engaged in today. This effort might include letters, article submissions, personal meetings, and, yes, even lobbying.
2. If the editor were still non-responsive, either figuratively or literally, the aggrieved reader would next communicate with the journal’s ombudsman, then with its publisher, pointing out the lack of adequate response or redress of uncorrected lapses of the sort we are discussing this morning.
3. However, in the unusual circumstance where these steps were ineffective, I would then personally neither submit to this journal, nor would I do any peer review for it.
4. Finally one could lobby friends and colleagues to *shun* (but not formally boycott) such a journal.

I speak most resoundingly for the resolution which I will now repeat for clarity:

A medical publication which promotes political agendas has no place in scientific and medical journalism, and academics should refrain from publishing in such journals.

In conclusion, academics with important scientific contributions should shun (but not formally boycott) a journal which acts in a biased, one-sided manner, and which distorts its scientific mission, especially when it abuses this mission for purely partisan political reasons. I would strongly encourage serious medical scientists to submit elsewhere, just as I would recommend to the next Picasso not to choose to hang his works in a gallery that did not reflect his artistic values.

## CON STANCE: RAEL D. STROUS, MD

What is freedom of “*academic*” expression? Without the freedom to offend, it ceases to exist.

(Salman Rushdie)

Mr Chairman Sir, ladies and gentlemen, I am not here today to extol the virtues of anyone’s political views, perspective, or *Weltanschauung* (worldview). I am not here today to wax on lyrically, relentlessly, and mellifluously over the merits or demerits of any military campaign or international injustice perpetuated by any government or global plot against any one particular population or religion—even though by the end of what I have to share, you may have some idea of where I stand on these matters.

I am, however, here today to speak about freedom of speech in general, about academic freedom in particular, and about misunderstandings and clarifications regarding the concept of boundary violations among medical professionals and institutions, including medical publications. My aim today is to prove to you that medical publications which may promote political agendas do have a place in scientific and medical journalism. However, and perhaps most importantly, I am here to state *categorically and unequivocally* that even if you do not agree with me on this last-mentioned point, academics and medical professionals should emphatically *not* refrain from publishing in such journals.

### Issue One: *Medical publications which may promote political agendas do have a place in scientific and medical journalism*

Medical professionals are the recipients of respect that is due to them from society by virtue of the unwritten social contract that the community has with doctors. Thus, physicians are allowed to undress patients, make deep body incisions with knives, remove organs, and probe the innermost intimate issues with patients during a consultation. This agreement is coupled with the duty to relieve pain and suffering and to manage disease and disability.^2^

It is imperative, however, that physicians maintain the boundaries of this interaction so that the patient/society can interact in a safe atmosphere with a physician who is completely in sync with the patient and clear of any presumptive political orientation or grandstanding. Any political barrier that is placed between the two interferes with the special and unique interaction owed to the patient. Physicians need to desist from involvement “as physicians” in areas that supersede medical practice and refuse to employ their training and professional skills in areas where they do not belong, such as torture as defined in the Declaration of Tokyo. This would apply as well to physician assistance in interrogation, profiling, electoral candidate character assassination, or anything else unrelated to the purpose for which the doctor underwent medical training.^3^,^4^

But—and this is a big but—physicians can and should be involved in aspects of the political process; however, this should only be in order to obtain better conditions and resources for their patients. Furthermore they may assist in engendering better education of the public with respect to illness prevention and optimal treatment as well as insisting on equality of health care for all.^3^,^5^ Gruen and colleagues^6^ have referred to such a role as “advocacy for and participation in improving the aspects of communities that affect the health of individuals.” Several examples of such socio-political involvement exist over the past century, including the celebrated Polish doctors in the early 1900s.^7^ Similarly, during the Nazi era, doctors had this duty; unfortunately, only on rare occasions did they speak out against the atrocities extended to the mentally ill. The mind boggles at the thought of what might have been, had the physicians during that era spoken out; remember, it took close to two years to halt the gassing of the mentally ill—all before the Holocaust “officially” began—and only after a very few individuals spoke out against the process. Although Hitler had given permission to physicians to kill their patients, he officially stopped the program to gas the mentally ill, which had been ongoing from 1939 to 1941. Hitler himself never ordered the process!^8^ Physicians *need to speak out* in the context of medical publications on issues they feel to be of profound relevance to public health and well-being.

Another way of understanding this process is by considering the interface of facts and values. An archaeological or physics journal deals with the facts only—it has to by nature. A medical journal, however, is different; it deals with well-being, values, virtue, interaction, sociological phenomena, and cultural competence. This is by nature what is of importance to medicine—this is what is demanded today from a medical journal in order to inject more of an emphasis on virtue in the field. One may consider that this is in keeping with a “post-modern” approach to academia and information, which is predicated on the presumption that not everything is “factual,” rather all is subjective. The question arises in medicine whether it is in fact possible to separate facts from values. From the perspective of Thomas Kuhn, this is impossible. Even if it were possible to separate facts from values, is this what we want in medicine? Is this what we want from a flagship international journal of the medical field? It is imperative that a medical journal appeals to virtues and open discussion of issues even if uncomfortable and confrontational to some. I argue, however, that facts need to be facts and virtues need to be virtues; it is not “anything goes.” That is how we develop as discerning doctors, by learning and gaining the skills to know what are facts and what are authentic values, and to develop a *Weltanschauung* for ourselves in the field. I implore my opposing speaker: Please don’t limit me on that.

Mr Chairman Sir, refraining from publishing in any particular publication weakens the merit of values; boycotting removes values from the field even if the absence of such rash measures opens the door to vastly different opinions. Dialogue is essential to the field. I ask you: did anyone seek to boycott the *Journal of Medical Ethics* after it published what many considered to be an extremely offensive article justifying “after-birth abortion?”^9^ No, Sir! Instead, it affords me with the opportunity of matching their non-factual opinion-based argument with a dialogue on virtue and values that trumps their approach. I succeed in both accounts— I combat their view, I introduce debate on a high level around the value of life and the physician’s responsibility, and I relish and bask in the glory of a civilized debate over issues of critical importance to society and medicine.

I would ask you, ladies and gentlemen, is there not in fact a parallel between opposing articles of vastly differing opinions existing side by side in a journal and the profound contrast, well known to Israeli medicine, where emergency rooms and hospital wards commonly have terrorists and terror victims lying side-by-side as recipients of the identical state-of-the-art medical standards and attention so characteristic of Israeli medicine and values? Should we not entertain similar values and ethical discourse in the medical literature? It is the conscience of the doctor in medicine that is important—we do this by encouraging dialogue and at times even challenging the status quo in society regarding issues of relevance and importance to public health in policy and well-being. Who would not have wanted able-bodied confident doctors to speak out in 1939 against the policies of the German T4 program regarding “life not worth living” and the subsequent euthanasia of approximately 200,000 mentally ill people. No doctors spoke out in the international literature to any significant extent, although some did in the USA, albeit to support much of the theoretical underpinnings of the program.^10^ How apt are the words of the great American poet and author Ella Wheeler Cox: “To sin by silence, when they should protest, makes cowards of men.” It should be remembered that it was the British, the Canadians, and the Americans who were at the forefront of the eugenics movement and not the Germans. However, it was the German doctors who actualized the eugenics program based on synergistic goals of the medical professionals and broader political forces. How different history may have looked had physicians openly protested in the medical literature regarding the euthanasia program—on which many would argue the Holocaust was based.

I therefore want to go above and beyond politics. Medical publications need to address fundamental concepts including issues of conscience and virtue. This even includes trying, at times, to cultivate a healthy skepticism towards our own points of view (and that, ladies and gentlemen, is a direct quote from the written words of our very own chairman of the debate, Professor Glick, in a 1994 paper he authored and published in the *Journal of Medical Ethics*).^11^ This is an example of an issue that is more than “just” about me as an individual or me as an Israeli doctor. Einstein once quipped: “Try not to become a man of success, but rather try to become a man of value.”

Mr Chairman Sir, I am not asking you to agree with any partisan political rhetoric of any particular medical journal. I am not asking you to condone any falsehoods, inaccuracies, offensiveness, etc. However, I *am* asking you to agree that medical professionals have the ethical right to stand up and make an issue of public health where they may feel one exists. If they do so, I submit to you that this *has* to be carried out within the context of a medical infrastructure such as a conference, journal, or institution and not through traditional political structures. To carry it out through political structures would be a boundary violation and considered an egregious infringement of medical ethics and professionalism. However, addressing a medical journal with a problem affecting public health is a duty of medical professionals as described above.

I would, however, agree with Daniel Patrick Moynihan that “everyone is entitled to his own opinion, but not to his own facts.” In the words of Chesterton, “to have a right to do a thing is not at all the same as to be right in doing it.” As Hubert H. Humphrey so aptly put it, “the right to be heard does not automatically include the right to be taken seriously.”

Herein lies the crunch—do not fear taking on such viewpoints. Do not fear exposing opposing views in the medical literature, whether they are with a political stance or not, for what they are—be they factually, ethically, numerically, statistically, or philosophically incorrect. Samuel Johnson once remarked that “every man has a right to utter what he thinks truth, and every other man has a right to knock him down for it.”

It may be that you, like me, do not want to be associated with such people, but I also don’t want to limit their speech in any way because that’s one of the things freedom requires. As Rand Paul once quipped, “even if we allow people to be boorish and uncivilized, that doesn’t mean we approve of it.” Bryant McGill said, “Do not make the mistake of thinking that you have to agree with people and their beliefs to defend them from injustice.”^12^ Take them on at their own game. However, this is very, very different to that very dirty “B” word: *boycott*.

### Issue Two: *Medical professionals should emphatically not refrain from publishing in such journals*

And this brings me to my second point as elucidated earlier on—even if you do not agree with me that medical publications with political content have a place in medical journalism, I wish to prove to you that medical professionals should emphatically *not* refrain from publishing in such journals.

It is generally accepted as an axiom that the progress of science and knowledge is dependent on the open exchange of ideas between academics.^13^ An official statement of the principle has even been formulated for scientists by the International Council of Science (ICSU). Statute 5, referred to as the “Principle of Universality of Science,” clearly states:

The Principle of Universality (freedom and responsibility) of Science: the free and responsible practice of science is fundamental to scientific advancement and human and environmental well-being. Such practice, in all its aspects, requires freedom of movement, association, expression and communication for scientists, as well as equitable access to data, information, and other resources for research. It requires responsibility at all levels to carry out and communicate scientific work with integrity, respect, fairness, trustworthiness, and transparency, recognising its benefits and possible harms.

In advocating the free and responsible practice of science, ICSU promotes equitable opportunities for access to science and its benefits, and opposes discrimination based on such factors as ethnic origin, religion, citizenship, language, political or other opinion, sex, gender identity, sexual orientation, disability, or age.^13^

Thus, the open values of science encourage debate and discussion on the way to the truth and preclude any form of embargo or sanctions based on the opinions of others. Mr Chairman Sir, this could not be stated more clearly!!

This Principle of Universality of Science clearly stands in tension with the practice of academic boycotts of individuals or journals. An academic boycott embraces the “systematic withholding of normal professional relations from academics as a means to achieving some goal, typically either punishment or the bringing about of some change in behavior or policy.”^12^ Many examples of boycotts of Israeli academics exist around the world, including the Annual Congress of the University and College Union (UCU) in the United Kingdom in 2007. And now, Mr Chairman Sir, the proposing speaker today is suggesting we do the same. Is the very respectable professor from Beer-Sheva aware of what he is proposing? Do we not hold ourselves up to a higher standard of ethical norms?

May I submit to you that boycotting a medical publication based on its publication of relevance to public health despite overt political content would directly conform to the moral *impermissibility* of academic boycotts.

Professional discrimination is unfitting when it is based on deliberations extraneous to ethical principles and quality standards such as political factors and affronts to one’s sensitivities. Open dialogue, on the other hand, is demanded, and it is there that such an offense or even outrage needs to be confronted and purged.

The Principle of Universality is based on two considerations: the extent to which universality contributes to the value of science and academia, and the rights of academics and their institutes, including journals, to be free from inappropriate methods of discrimination.^14^ Thus, boycotting an academic journal based on its publication of literature deemed to be inappropriate, no matter how offensive, would be downright unethical and in direct contravention of the ethical principles of scientific endeavor and professional values of medicine.

Advances in science and learning are essential to society and its well-being. A critical aspect of this fundamental good is that it is social and collaborative. Hence, academic endeavor by definition inculcates, as crucial features, interaction, communication, and diffusion—a phenomenon that boycotting comes to obliterate. It is true that at times there are publications that may be accused of falsification of facts, poor science, political boundary violations, etc. However, to take a stance on boycotting every time a publication of such ilk sees the light of day, as the proposer states, would be the height of idiocy and misguided vigor. Boycotts are proscribed because they diminish or destroy the value inherent in science and learning. Boycotts deliberately obstruct interaction and cooperation, and therefore establish impediments to scientific advancement.^14^

There is, in addition, a further aspect to consider regarding the dangers inherent in the significant risk of the exploitation of any boycott. Since no benchmarks for the absolute rationalization for boycotts exist, any boycott may be exploited as an example by questionable individuals or organizations to back other politically motivated and groundless boycotts. In this manner, supporting or justifying any boycott complicates the quest to combat unwarranted and damaging boycotts in other situations.^14^

As both Jews and Israelis we have been the victims of such injustice in many such circumstances. For example, I can point to Nazi Germany and the contemporary Boycott, Divestment and Sanctions Movement (BDS Movement) as such examples in the academic arena!

Furthermore, why is refraining from publishing in any particular journal so problematic in humanistic terms? It is because the long-term consequences of publishing any particular academic work cannot be known. Thus, if Israeli researchers, albeit limited in number, decided to abstain from academic interaction in the international arena such as certain prominent medical journals, this might result in undue consequences for medical and/or academic advancement. Even if the boycotting of a journal by Israeli academics would lead only to postponement of a breakthrough (theoretically a possibility), this would still be unethical due to potential effects on well-being for some.

I would like to propose, as the scholarly and eminent Rodin and Yudkin^14^ have before, that there are three tests to be used when judging whether the probable welfare contribution of a proposed boycott is sufficient to outweigh the harms and risks, and whether or not the boycott is likely to be justified according to ethical and professional norms.

First, a boycott can only be acceptable if there is a good chance that it will succeed in combatting the moral malevolence at which the boycott was aimed. If not, the boycott fails to meet the standards of the Principle of the Universality of Science discussed earlier, with resultant consequences, for no matching advantage. Mr Chairman Sir, ladies and gentlemen, do you really believe that an academic boycott by Israeli academics of any medical journal, be it the *New England Journal of Medicine* or the “Ugandan Journal of Snow Science” would have any affect? Even if you suggest that publication of anti-Israel rhetoric is clearly masquerading as modern-day anti-Semitism in disguise, and that all Jewish academics around the world should shun the journal—do you really think that we could muster up an international cabal of Jewish scholars with the support of all the elders of Zion and their protocols, and that this would have any influence on ingrained anti-Semitic international Jewish control conspiracies?

There is absolutely no evidence that prior academic boycotts have significantly contributed to the dissolution of serious iniquities. Many have referred to the example of the academic boycott of my home country, South Africa. Most would argue that the academic boycott in this case did nothing to enhance the end of apartheid. On the contrary, as noted by Rodin and Yudkin,^14^ it may have contributed to what Neville Alexander later termed “the scholarly backwardness of South Africa today.”

The second moral argument that a boycott must satisfy is that it be necessary and that there is no other alternative that could realistically project the preferable outcome with less moral expenditure. If other approaches are available to attenuate the “moral evil” such as dialogue and debate, then these should be followed, rather than the option of boycott. Boycotts should not be warranted until other more reasonable approaches have been exhausted. In this manner, boycotting should be left to the auspices of state entities, not academics or scientists.^14^

Since the fundamental importance and significance of academia and knowledge is so profound, and the consequences of interfering are so stark, the third moral argument of Rodin and Yudkin states that a boycott could be vindicated only if it is an “exceptional response to a grave evil.”^14^ If it would be decided to engage in boycotts in unremarkable situations, the complete organization of collaborative science and learning would be compromised. Furthermore, universalizability demands an ethical requirement for academic boycotts in every circumstance in which the defining principles for a boycott exist. Hence, any country with any form of considered injustice, no matter how severe the injustice, needs to be boycotted. This would destroy the whole fabric of academic dialogue and interchange of ideas. An important practical corollary to this suggestion is that it is *ethically problematic to* boycott against ethical violations of less significant consequence whilst desisting from boycott against evils of a much greater level. Is it more important to boycott a medical journal publishing an incendiary political piece, than an academic journal from a country planning to drop a nuclear bomb on Israel? Where do you draw the line?

Mr Chairman Sir, may I submit to you that the academic boycott of a medical journal fails on all three of these ethical standards despite the potential egregious nature of published material in such a journal.

Boycotting locks in the biased article—with no commensurate response in the offing. Publications often make errors—they need to stand to be corrected not boycotted. I ask you, would it be wise to boycott the *New York Times* after it published an incorrect article about crack babies? Would you boycott *Time* magazine after it erred and falsely accused Ariel Sharon of planning the Sabra and Shatilla massacre? Even though the latter magazine was proven wrong in court, it stubbornly refused to retract. Boycotting, however, closes off the possibility of correction and betterment of important sources of knowledge.

Thus, given that boycotts are a deviation from well-grounded moral principles (as I have argued), the burden of proof should reasonably fall on those who propose and support boycott measures. Based on the opening argument of Professor Clarfield today, such measures are clearly unfounded under the circumstances.

### Summary

My aim today is not to discuss the merits or demerits of any particular published piece of literature in any medical journal. Rather my goal is to reaffirm the value of open debate about critical topics that, although politically charged, entail profound effects for health around the world. Medical journals need to address issues of value even if in so doing they challenge the sensibilities of some and fail in their aim of fostering virtue in the field. Attacks on journals for publishing authors’ concerns on issues of public health with political undertones threaten the integrity and independence of all scientific journals dealing with medicine and community health in general, and values and virtue in the medical field in particular. As John Howard once quipped, “It’s too much to expect in an academic setting that we should all agree, but it is not too much to expect discipline and unvarying civility.” It is also not too much to expect unwavering professionalism and a commitment to profound tolerance and forbearance.

I thank you.

## RESPONSE: RICHARD HORTON, MD

I certainly do not wish to give a long speech, but I would like to add a few words if I may.

The first thing I would like to say is thank you for holding this debate, because I believe it is in the highest standards of academic discourse to do so. It is a great tribute to you for 1) having the courage to invite me to visit your nation, and 2) holding a debate such as this, where we can discuss in a serious intellectual way an issue that we all agree concerns a very difficult aspect of medical science and ethics.

Now, if you will forgive me, I will respectfully disagree with Mark! This resolution is in two parts and it is important we separate those parts—first, journals that promote political agendas and, second, academics who should refrain from publishing in those journals.

On the matter of political agendas, let me try to clarify the meaning of the words we are using. The word “political” has a very precise meaning. It means “relating to the government or public affairs of a country.” That is all it means. An “agenda” means, “things to be done.” Are we really saying that medicine is unrelated to government actions or the public affairs of a country? I come from a nation where the idea of a National Health Service (NHS) was founded in the wake of World War II as an expression of national solidarity directed towards a very clear objective—health equity. The notion of health equity at the center of our political discourse is creating problems for us at the moment, so embedded is it in our national consciousness, our national culture. For many doctors and a large part of the public in the UK, the NHS is something to fight for, defend, and believe in—it’s a deeply existential and political matter. The politics of the NHS are inseparable from the ordinary day-to-day practice of medicine in the NHS. As we approach an election in 2015, the politics of the NHS will be at the forefront of our national debate.

Should we, as doctors, or should medicine or medical journals not take part in this debate? Should we not have our own political views and priorities about health? Should we simply say, “Well, it’s up to you in government to decide about health, and we doctors will be your servants. You make the political decisions, and we will implement your decisions.” Or do we not feel, as health professionals, that we should have some stake in that political debate ourselves, that we should make our own political arguments about what might be done?

If you put those two ideas together—first, that there are issues in politics, public affairs, and government which relate to medicine and, second, that health professionals are entitled to an opinion about those issues—it seems to me impossible to say that medical journals should not have a political agenda. Indeed, I would turn it around and suggest that it would be an outrageous dereliction of our responsibility as health professionals if we did not have political agendas. One of the great scars across scholarship today in the sciences, and perhaps the medical sciences in particular, is that we have failed to embrace a political agenda, to stand up for a set of values that we as a profession believe in.

Let me turn to the criteria that Mark set out for a meaning of “fair and unbiased.” What is “fair and unbiased”? Take the issue of MMR—the debate from some years ago now about the safety of the measles, mumps, and rubella vaccine. The media interpreted “fair and unbiased” to mean that whenever there was an interview, you gave five minutes for those who thought the vaccine safe, and five minutes for those who thought the vaccine unsafe. This definition of “fair and unbiased” can carry within it hidden harms that can cause huge damage. So I think one should be very careful about simply accepting the idea of “fair and unbiased.” Indeed, sometimes there will be moments when reasonable people will want to make a judgment—to consciously not be unbiased—and to choose a particular side. Our commitment should not be to say, always, on every issue, that a debate should proceed for the point of view of “on the one hand this” and “on the other hand that.” The commitment should be that if one is going to take sides, as sometimes may be necessary, then one opens one’s publication up to a full and open debate after you have taken that particular side. The much more serious criticism would be if we published a piece that took one side of an argument and we then closed our doors to critics.

Should we therefore not promote, as Mark said, a political agenda? I have mentioned the NHS already, but there are many other areas in health and medicine where we should most definitely be promoting a political agenda. Each medical journal will have its own political concerns. That is good for pluralism in medicine. Our agenda is very much centered on global health. We have actively promoted political agendas in relation to, for example, international aid and laws that hurt particular communities (e.g. laws promoting homophobia or laws against sex workers). We see these political standpoints as part of our duty to promote political reforms that advance health.

Let me turn now to the issue of conflict of interest. Conflict of interest has become, in Ken Rothman’s words, the new McCarthyism of science. It is easy to label people as a way to excuse not engaging with their argument. Surely we can do better than this as a medical community. If a writer poses an argument relevant to health, let us engage with that argument. One person’s conflict of interest may well be another person’s expertise or experience. We once had a policy at *The Lancet* of not using statistical reviewers who worked within the pharmaceutical industry. But one particular statistical reviewer disagreed. He argued, “So you are saying that simply because I work in industry, I have a conflict of interest that renders my scientific opinion worthless?” We had to admit that our policy, as well intended as it was, was wrong. We are very careful now about letting critical personal labels obscure good arguments.

On the subject of a boycott, the resolution was, “Academics should refrain from publishing in such journals.” The definition of a boycott is “withdrawing from relations with an entity as a punishment or protest.” Many of my friends who work in the occupied Palestinian territory support the Boycott Divestment Sanctions Movement and ask me if *The Lancet* would support that Movement too. My position is that we would never support such a boycott. Boycotts are quite simply wrong. In my view, the right way to achieve any kind of change, irrespective of one’s political agenda, is to open up a channel of communication with those you disagree with. Only through communication and talking and sharing experience and knowledge can we expect change to happen. So we must resist, indeed we must condemn, boycotts in every possible way. Communication is the best and only constructive way forward. I agree with both of our speakers when they emphasize the importance of dialogue. Indeed, I am here in Israel to engage in such a dialogue. Although I have only been here just over 24 hours, I have already learned a great deal, and I believe we can turn this experience into something very positive in the long term.

I will end now, if I may, with two quotes. The first is this:

Remaining neutral in the face of injustice is the hallmark of the lack of ethical engagement typical of docile populations under fascism.

The second quote:

Health workers should not stand by while injustice leads to the death and injury of civilians in the conflict that could be prevented.

Who wrote these sentences? They are Jewish health professionals living in South Africa. They wrote these words at the end of August, 2014. And they wrote them as witnesses to the worst excesses of state brutality under apartheid. When they were living under apartheid, they desperately wanted a forum to express their views about the political regime in which they were working. They had no opportunity, no forum, to express their views. We should be glad there are places where those who feel injustices exist can express their views freely and openly—and, of course, accountably.

I am going to make a plea to you: political agendas are something to be encouraged, supported, and promoted in the very best interest of our patients and those populations whom we serve. We should not only not refrain from publishing in journals, but also we should engage with journals even more strenuously when we disagree with them, as you have shown by inviting me here this week. I promise you that by engaging with those journals, their editors will listen and their views will change. You will educate them, enable them to choose a different path, and, through that process, create opportunities for a better and more hopeful future.

I wish to thank our two speakers, our Chairman, and you, the audience, for allowing me this opportunity to say these few words. Thank you very much indeed.

## PRO REBUTTAL: A. MARK CLARFIELD, MD, FRCPC

First, thank you all again.

Professor Strous, I have been most impressed with both the passion of your argument and with the clever choice of some of your examples. That being said, it does seem to me that you have transmitted a bit more heat than light in your strenuous efforts to speak against this morning’s most reasonable resolution. Furthermore, I think your logic is actually faulty in some cases.

Of course all of us are in favor of freedom of speech. I don’t think there is any argument about that; and, as our audience will attest, I have not this morning mentioned anything about interfering with such a sacred liberty. That being said, all sensible people know there are also limits to all rights, even freedom of speech. No value is absolute. Taking such a right to an illogical conclusion as you have done so skillfully today can not only be frivolous but dangerous. I therefore suggest, with respect, that this argument, at least in the unidimensional way you present it, is not all that helpful.

Also, it seems to me that you took the liberty of rewriting the resolution. I would like for clarity to repeat the proposal over which we are having such a spirited contest; to wit: “A medical publication which promotes political agendas has no place in scientific and medical journals, and academics should refrain from publishing in such journals.”

I do not see anything in the resolution about boycotting. Although the resolution did not mention it specifically, I know that the word “boycott,” like the proverbial elephant in the room, is there—and so I addressed it. I tried to make it perfectly clear that I am against the idea of boycotting. However, I do think that it is the right of potential authors to avoid submitting to a publication that breaks accepted norms of academic and intellectual fair play; as addressed in the second part of the resolution: “… academics should refrain from publishing in such journals.”

Are you suggesting that I should seek out a journal which I think is acting unfairly and try to publish in that publication as a means of supporting free speech in medical journalism? Or do you not believe that an author has the right to pick the journal to which he or she wants to submit and to make value judgments on various levels about such journals?

If you will permit me, I would like to return to the word “shun.” Indeed I had wondered about the advisability of using this term; perhaps “avoid” is less contentious, but the intention must not be masked—I’ll leave that choice open, but I would like to know what the members of our audience think.

Also, you mention the role (or more specifically the *lack* thereof) of German doctors during the benighted Nazi period of European history as a kind of epic saga which you posit supports your claim for absolute freedom of speech. In other words, if I understand you correctly, had more German doctors outside of their professional expertise courageously protested against Nazi policies, perhaps history would have taken a different course.

Unfortunately, whenever we have an ethical debate, not just in Israel but in many places, all too often we go back to our old enemies, the Nazis. It is true that they really do represent the very epitome of evil and it would be hard to argue that in the good old days of the Nazi journal, *Der Völkischer Beobachter*, doctors should not have protested against the publication of such racist cant. I am certainly not saying that any of the material we are talking about today nor the journals to which we may be referring this morning come anywhere close to that. I will not use that as an analogy.

Of course the doctors in Nazi Germany, as well as every right-minded citizen without an MD, had an obligation to fight politically against the Nazi regime. It has everything to do with them as people, as good citizens, but not primarily as doctors, nor with their medical expertise. As *people* they had a role and a responsibility to fight that horrid regime in every way they could. As doctors, those (many) who eagerly joined the Nazi party distorted and perverted their work by what they did and, by so doing, poisonously politicizing medicine.

That is exactly what I am afraid happens if we do not follow today’s resolution. Not that we become Nazis of course, but that medicine as a practice and physicians as its practitioners become overly politicized and we start having political debates about something that is primarily a medical question or even worse, vice versa. Hence, Professor Strous, I would like to ask you directly: do you really think that research, for example about global warming, comparing the cost-benefit of coal versus natural gas belongs in the pages of the *New England Journal of Medicine*? Is that the place to discuss such a technical question? Or is that the place to discuss global warming as a threat to human health, but to defer to our engineer friends with regard to the cost-benefit of energy alternatives and say, “Tell us something please. As physicians we know that global warming affects human health in the following ways, and that it is a very serious concern; but what does society need to do to lower carbon emission?”

This is not a medical question now. But global warming is indeed one of the most serious potential threats we face, I would argue—apart of course from an errant asteroid hitting the Earth, which you might be surprised to learn actually is certainly well within the realm of possibility. It has happened before. But to take my argument even further, one could with some justice argue that we are not investing enough in avoiding asteroids, and maybe some concerned physician should write something to a medical journal about this danger because it could actually destroy all life on this world. But if you really wanted to stop an asteroid from hitting the Earth, if you chose to argue in a medical journal that we need to develop more robust anti-asteroid defenses—and I guess we do—you would then not open a debate in that medical journal about the best way to knock a heavenly body out of the sky. Nor would you have an argument in that publication about whether fracking was more or less expensive, or polluted water, or was more costly than other forms of energy extraction. You could certainly write an article on the effect (or lack thereof) of fracking on human health, but not one which explored the technical sides of this complex geological issue.

So I conclude, interestingly enough (and in the end not completely surprising given that we are both reasonably intelligent people and you, my worthy opponent, are *very* intelligent), that I am actually in violent agreement with much, albeit not all of what you have said this morning. And it is over this small difference that I have so enjoyed crossing rhetorical swords with you today.

## CON REBUTTAL: RAEL D. STROUS, MD

A journal sometimes must take risks and break new ground in order to stay abreast of complex intersecting fields. By giving voice to those who have strong opinions on issues of public health and related topics, even if controversial, the journal is addressing virtue in the field and thus has advanced dialogue. We have all learned, been nurtured, and have gleaned much from the annals of medical journals. We cannot embargo according to our whims. I would expect more from my esteemed colleague and opponent from the *city of seven wells* down south who should be more acquainted with the well-known adage from the Talmud (Tractate Baba Kamma 92b) that we “do not throw stones into a well from which we have drunk.”

Whatever you may think of any medical journal publishing varied points of view, even if they may offend your individual sensibilities, you have to respect the journal for allowing diverse points of view to be discussed in an independent academic space when they have relevance to matters of public health. Thus, Mr Chairman Sir, to paraphrase Peggy Noonan, “We don’t need to ‘control’ free speech, rather sometimes we need to control ourselves.”

It appears that my opponent has failed to understand the fundamental difference between a soapbox and an academic medical journal. An academic journal should, and must, take positions related to public health which are morally obvious, clear, essential, and urgent—and they should, and must, avoid opinions that are partisan, arguable, and morally ambiguous.

Partisan argument is properly worked out on the soapbox, in the speaker’s corner, and on the op-ed pages of newspapers—these are the spaces that allow for argument, banter, wrong, and right opinion clashing. They are boxing rings. I would agree with my esteemed opposing colleague that this should not be the case with prominent medical journals of standard, which is a place where the only statements that ought to be made are ones reflecting scientific and moral truth, not scientifically and morally arguable opinion.

However, what is a moral truth, or a morally clear position? It is a moral position which is held by the great majority of the moral community when it is well informed—several examples of which include opposition to Nazi racial policy or to Soviet psychiatric practice for hospitalizing dissidents. The great majority of decent scientists would take deep moral offense at these positions.

What is a partisan moral position? It is a position about which the moral community is undecided, even though it is reasonably informed. Partisan positions often reflect bias or loyalty to one of the parties of the conflict, or abidance to a particular and arguable ideological position (i.e. Zionism is a form of colonialism), which colors the moral reasoning. They are inconsistent with John Rawls’s principles of fairness and justice as elucidated in the Law of Peoples and Theory of Justice, which requires that moral reasoning be disconnected from side-taking.

A medical journal should not take a partisan position. I would agree with Professor Clarfield that in the case we are discussing, the journal in question perhaps did err. It appears to have hid the opinions and backgrounds of the article’s writers because it understood that they themselves were biased and partisan, and hence the moral arguments presented in their article were biased and partisan. Publishing letters of response is not optimally palliative, as it is viewed by the readership as a “necessary concession” to Jewish and Israeli readers, whereas the article is published by editorial *fiat* and hence representative of the editorial board point of view. It is hoped that a good editor would recognize such an error and address it accordingly, if deemed to take a partisan rather than moral stance. Alternatively, an editor or editorial board will demand correction of factual errors especially when the good name of a flagship journal of medicine is at stake.

However, even if one may argue that *The Lancet* behaved as a soapbox, not as an academic journal, the question is what to do with this. Refraining from submitting or boycotting as I have emphasized is not the solution!

It was George Orwell who once quipped that “In real life it is always the anvil that breaks the hammer …” If you consider that some prominent high-impact medical publications, as a result of what they publish, are more like soapboxes than journals—then tell them so. Do not lock in the improprieties by refraining from publishing. Do not flatter the editor with the inevitable attention that he or she will invoke from much of the world’s biased, anti-Semitic-leaning rhetoric. Stand up for your values and show up the journal by responding in whatever format you can; if the good standing of the journal suffers for publishing biased trivia, or the impact factor plummets—then so be it. As Phillip Sharp once stated, “the right to free speech and the unrealistic expectation to never be offended cannot coexist.”

Hence, it is not retraction, as my opponent demands, but discussion, deliberation, and argument that advances civilization. Regarding the current conflict in Gaza, it may be argued that the views of medical associates contribute to engendering options for the well-being of innocent citizens on both sides.

Noam Chomsky once commented that “Goebbels was in favor of free speech for views he liked. So was Stalin. So, my esteemed colleague, if you’re really in favor of free speech, then you’re in favor of freedom of speech for precisely the views you despise. Otherwise, you’re not in favor of free speech.”

Let me summarize with the following words of the prominent social reformer William Lloyd Garrison: “I am aware that many object to the severity of my language; but is there not cause for severity? I will be as harsh as truth, and as uncompromising as justice. On this subject, I do not wish to think, or to speak, or write, with moderation. No! No! Tell a man whose house is on fire to give a moderate alarm; tell him to moderately rescue his wife from the hands of the ravisher; tell the mother to gradually extricate her babe from the fire into which it has fallen;—but urge me not to use moderation in a cause like the present. I am in earnest—I will not equivocate—I will not excuse—I will not retreat a single inch—*and I will be heard*.”

I implore you, ladies and gentlemen, do not allow the exuberance of your misplaced endeavor to be the death of your intended ethical principles and professionalism. Do not allow articles that bother your sensibilities to affect your judgment of the bigger picture. Rather, respond appropriately. Einstein once said: “In the middle of difficulty lies opportunity.” I beg you to invoke the power and virtues of peace and justice in determining your path in academia. Be ruled by your mind and not your heart, no matter how well intended your ethical sensitivities.

As a student of the teachings of the great Jewish physician, Rambam, colloquially known as Maimonides, the pillars of knowledge are defined not by “thinking the truth,” but rather by “knowing the truth.” When you read a piece of medical literature and you *know* it to be wrong, do not play the perennial victim and cry foul; go for the jugular—attack it for where it is at fault, then counter-attack. Show the world how pathetic it is and where editorial judgment is “out of whack,” but do not question the right of individuals to publish what they feel to be of importance from the perspective of public health. A medical journal should be virtue- and value-oriented, at times including narrative which is not only “factual based” by strict definition. That is in keeping with our social contract with society. A journal should publish on contentious issues of relevance to medicine such as immigrant health, contact with pharmaceutical companies, medical insurance policy, health inequalities, *and* behavior during war according to acceptable principles of respect for health care.

Acting out by means of boycotting or refraining from publication in any journal locks in the error with no recompense for correction. There are very rare extra-special circumstances, under cases of clear moral outrage—unambiguous and internationally mandated, where there may be place for sanctions—but they need to meet certain well-defined criteria as previously delineated.

Our physician community devoted to scientific integrity, fact, and compassion should focus our collective principles on promoting health, safety, and security for all. If it means that at times we need to turn to the written word, albeit controversial at times, then so be it.

I will end with the forever powerful words of Voltaire: “I may not agree with what you have to say, but I’ll defend to the death your right to say it.”

I thank you.

## DEBATE CLOSING: SHIMON GLICK, MD

Thank you, gentlemen, for two stellar performances that were vigorous, forceful, and almost convincing. I envy your rhetorical abilities and am happy that I did not get the difficult task of choosing a winner.

I will now violate the classic rules of debating societies and take advantage of holding the microphone and give you my personal views on this controversial subject.

We live in an era in which medical schools and other organizations are being asked for social accountability, a demand with which I agree whole-heartedly. There are undisputable data from all over the world that health is not a function merely of provision of medical care. We know that the major factor in ill health in every society is poverty, and obviously also military actions. *The Lancet* reported several years ago that three hours of world military expenditures would be enough to eradicate completely eight infectious diseases.

Decisions affecting poverty and war are clearly political decisions. Therefore I feel that not only is there a place for political agendas to be discussed in general medical journals like *The Lancet*, but there is an obligation for general medical journals to express and promote certain political agendas which have impact on health. This viewpoint was expressed so eloquently by Dr Horton last night.

But politics too has to meet ethical standards. It is not an oxymoron to talk about ethical politics. There is clean politics and there is dirty politics. Just as there is a clear standard for the quality of research articles, there must be a no less exacting ethical standard for political agendas. It is obviously even more difficult to define such standards in the area of politics. But there is no room for hidden conflicts of interest on the part of the writers, for factual distortions, for invective, and the like. Great care must be exercised not to descend to yellow journalism and careless evaluation of the situations which all too often characterize much of the non-medical media.

Now if a scientist or physician has an article which he is considering for submission to a journal in the face of an abundance of available medical journals from which to choose, I would imagine that he or she would, and should, prefer a journal which has a reputation for absolute integrity and fairness of editorial policy in all of its areas of endeavor.

From my personal knowledge of both of our debaters today I believe that they would agree fully with my summary, in spite of their overt disagreements for the purpose of the debate, and I have a feeling that Dr Horton too would subscribe to this position.

Thank you all again, organizers, speakers, and audience, for a most stimulating morning.

## Abbreviations:

CMAJ: Canadian Medical Association Journal;
ICMJE: International Committee of Medical Journal Editors;
ICSU: International Council of Science;
JAMA: Journal of the American Medical Association;
NEJM: New England Journal of Medicine;
WHO: World Health Organization.
